# CK2 in Cancer: Cellular and Biochemical Mechanisms and Potential Therapeutic Target

**DOI:** 10.3390/ph10010018

**Published:** 2017-01-28

**Authors:** Melissa M.J. Chua, Charina E. Ortega, Ayesha Sheikh, Migi Lee, Hussein Abdul-Rassoul, Kevan L. Hartshorn, Isabel Dominguez

**Affiliations:** Department of Medicine, School of Medicine, Boston University, Boston, MA 02118, USA; mel98791@bu.edu (M.M.J.C.); ceortega@bu.edu (C.E.O.); Ayesha.Sheikh@bmc.org (A.S.); migi0430@bu.edu (M.L.); habdulra@bu.edu (H.A.-R.); khartsho@bu.edu (K.L.H.)

**Keywords:** CK2, cancer, proliferation, apoptosis, migration, invasion, signaling pathways, signaling cascades, preclinical models, clinical trials, therapy

## Abstract

CK2 genes are overexpressed in many human cancers, and most often overexpression is associated with worse prognosis. Site-specific expression in mice leads to cancer development (e.g., breast, lymphoma) indicating the oncogenic nature of CK2. CK2 is involved in many key aspects of cancer including inhibition of apoptosis, modulation of signaling pathways, DNA damage response, and cell cycle regulation. A number of CK2 inhibitors are now available and have been shown to have activity against various cancers in vitro and in pre-clinical models. Some of these inhibitors are now undergoing exploration in clinical trials as well. In this review, we will examine some of the major cancers in which CK2 inhibition has promise based on in vitro and pre-clinical studies, the proposed cellular and signaling mechanisms of anti-cancer activity by CK2 inhibitors, and the current or recent clinical trials using CK2 inhibitors.

## 1. Introduction

There is strong evidence that CK2 plays a role in the pathogenesis of cancer [1,2,3,4,5]. Thus, CK2 functions as an oncogene when overexpressed in mice [2,4,5,6]. CK2 can regulate essential cellular processes, many of which are deregulated in cancer cells. In particular, CK2 increases cell proliferation [7,8], cell growth [9], and cell survival [10,11], changes cell morphology [12,13], enhances cellular transformation [4,5] and promotes angiogenesis [14,15].

**Figure 1:**
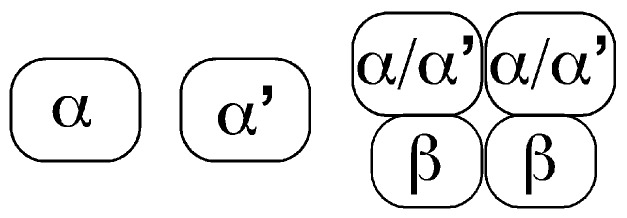
CK2 kinases can function as monomeric kinases and in a tetrameric complex.

CK2 kinases are a highly conserved serine/threonine kinase family, and in mammals, is composed of 2 genes: *CK2α* (*CSNK2A1*) and *CK2α*’ (*CSNK2A2*). CK2α protein has higher levels and more extensive expression in mouse tissues than CK2α’ has [16]. CK2 kinases can function as monomeric kinases, and also within a tetrameric complex (Figure 1). This tetrameric complex is composed of two CK2 units (CK2α and/or CK2α’) and two regulatory units (CK2β). The regulatory protein CK2β is coded by a different gene, *CSNK2B*, and within the CK2 tetrameric complex, it alters CK2 kinase substrate specificity [17]. In addition, an intronlesss *CK2α* pseudogene (*CK2αP*) can be expressed in mammalian cells and somehow it is relevant in cancer [18,19]. Importantly, CK2 proteins regulate each other’s levels. For example, knockdown of *CK2*α decreases CK2β protein levels, and knockdown of *CK2*β decreases CK2α’ levels [4,20,21,22].

As we will discuss in this review, *CK2* transcripts and proteins are upregulated in many forms of cancer. In some cases CK2 protein is increased without corresponding transcript level changes [23]. However, a recent study in five cancer types (lung and bronchus, prostate, breast, colon and rectum, ovarian and pancreatic cancers) found *CK2* transcript expression upregulated in some tumors, suggesting that transcriptional mechanisms may also play a role in the increase in CK2 proteins found in human tumors [19]. In general, CK2 transcript and/or protein upregulation correlates with worse prognosis. However, it should be noted that not all published data supports the general hypothesis that *CK2* gene over-expression is a driver of cancer progression and is associated with poor prognosis. One study found that some tumors show under-expression of *CK2* genes (e.g., CK2α’ in breast, ovarian, and pancreatic cancer), and that over-expression of the *CK2* gene correlated with higher patient survival in some tumors (e.g., lung adenocarcinoma) [19].

CK2’s ability to promote tumors in animal models may be largely due to its ability to regulate signal transduction pathways, which may vary in different cancers [24]. CK2 can regulate signal transduction cascades such as Wnt signaling [5,25,26], Hedgehog signaling (Hh) [27], JAK/STAT [28], NF-κB [5], and PTEN/PI3K/Akt-PKB [29,30,31,32]. Modulation of these signaling transduction pathways and cascades leads to tumorigenesis, indicating avenues that CK2 can induce cancer. For example, CK2 can activate Wnt signaling by phosphorylating and upregulating the transcriptional co-factor, β-catenin [25,26]. Indeed, β-catenin is upregulated in mice overexpressing *CK2α* in mammary glands [4]. CK2 may also promote tumorigenesis through stabilization of the proto-oncogene myc [33], activation of NF-κB, an anti-apoptotic factor in breast cancer [34], and inactivation of PTEN, a tumor suppressor phosphatase [31,32]. CK2 can inhibit Notch signaling in lung cancer cells and T cell acute lymphoblastic leukemia cells in vitro. This is particularly important since Notch1 regulates myc expression [35]. CK2 itself is also regulated by other tumor-promoting oncogenes, including Bcr-Abl [36]. Additionally, CK2 can downregulate the activity of tumor suppressors [37,38]. Moreover, CK2 inhibits DNA repair in some models, providing a rationale for combining CK2 inhibitors with chemotherapy agents that cause DNA damage (see cholangiocarcinoma trial). It is plausible that there are still unidentified additional biological effects of CK2 in cancer cells.

An ample variety of cell-permeable chemical CK2 inhibitors have been developed. The most frequently used are TBB, Quinalizarin, hematein, TBCA, CIGB-300, CX-4945, DRB, apigenin, DMAT, emodin, and TF [39,40,41,42,43,44,45,46,47,48,49]. We will discuss the use of these inhibitors in different cancers in vitro and in vivo, and the cellular processes and signaling pathways that they affect in each type of cancer. We will also discuss the two CK2 inhibitors, CX-4945 and CIGB-300, that have made into clinical trials.

## 2. Discussion

### 2.1. Solid Tumors in Which Over-Expression of CK2 Appears to Contribute to the Cancer Phenotype

Evidence is increasing that CK2 expression is linked to adverse prognosis in many common solid tumor types. These include tumors associated with chronic carcinogen exposure like non-small cell lung cancer, head and neck cancer, bladder cancer, or mesothelioma. CK2 is also involved in the pathogenesis of gastrointestinal cancers including biliary, liver, esophageal and gastric cancer, some of which may arise due to longstanding inflammation (e.g., hepatitis viruses for liver cancer or *H. pylori* for gastric cancer). CK2 is also linked to kidney cancer, HPV-related cancer (i.e., cervical cancer) and glioblastoma multiforme. As we review below, it is possible that CK2 contributes to these cancers in different ways. It is likely that initial trials of CK2 inhibitors will focus on patients with cancers that lack other effective treatments or are in advanced stages with recurrence after standard therapies.

#### 2.1.1. Environmentally Induced Cancers

##### Lung Cancer

Lung cancer is a leading cause of death in the United States. It can be divided into two broad categories—small cell (SCLC) and non-small cell (NSCLC) lung cancers. Numerous studies have shown the connection of lung cancer to smoking. Survival can be extended to some extent by chemotherapy in advanced or metastatic disease. The major clinical breakthrough is the emergence of FDA approval of immunotherapy agents like Nivolumab. However, more novel therapeutic approaches are strongly needed.

Rationale for CK2 inhibitors in lung cancers: *CK2α*, *CK2α’*, *CK2β* and *CK2αP* transcripts are significantly overexpressed in lung cancer [19,50,51,52,53]. Importantly, *CK2* gene expression is proposed as a prognostic marker [19,50]. For example, in lung squamous cell carcinoma, high *CK2α* transcript expression correlates with unfavorable prognosis for relapsed free survival, disease specific survival, and overall survival [50].

CK2 may play diverse cellular roles in lung cancer progression, including the control of cell proliferation, survival, migration and invasion. In vitro studies suggest differential cellular responses depending of the subtype of cancer. For example, CK2 inhibitors decrease cell migration and invasion in human adenocarcinoma and NSCLC cell lines, seemingly through downregulation of MMP-2 transcript expression and activity via the ERK pathway [54]. In SCLC and LCLC (large cell lung cancer), cell proliferation, but not cell invasion, is highly sensitive to Quinalizarin [55]. In contrast, in one adenocarcinoma cell line, invasion and apoptosis, but not proliferation, were sensitive to Quinalizarin [55]. In other adenocarcinoma cell lines, Quinalizarin only leads to increased apoptosis [55]. Hematein, a new inhibitor of CK2, also increases apoptosis in a human lung adenocarcinoma, corroborating the results obtained with Quinalizarin [41].

CK2 may play a role in metastatic lung cancer. For example, in adenocarcinoma cell lines, CK2 activity is necessary for TGFβ-1-induced invasion [56]. This effect correlated with changes in expression of EMT (epithelial to mesenchymal transition) markers such as *E*-cadherin, *N*-cadherin, vimentin, phospho-FAK (focal adhesion kinase), phospho-Src, Twist, and Snail, and MMP-2 and MMP9 [56]. In addition, CX-4945 inhibits TGFβ-1 induced-Smad2/3 activation and β-catenin activation, suggesting that CK2 is working downstream of TGFβ receptor activation [56]. Interestingly, TGFβ-1 significantly increases CK2α, but not CK2β, protein levels in the cytosol and the nucleus, and this effect is inhibited by CX-4945. This suggests that CK2 activity is required for the increase of CK2α protein levels promoted by TGFβ-1 [56].

CK2 may also be linked to stem cell maintenance through Notch signaling, which is found activated in lung cancer [35]. Thus, CK2 activity is sufficient and necessary for Notch reporter and gene target transactivation in adenocarcinoma cell lines, at least in part due to effects in Notch1 protein levels [35]. 

Integration of CK2 inhibition into treatment of lung cancers: The different cellular effects of CK2 inhibitors in different lung cancer cell lines indicate that CK2 inhibitors could be specifically used in particular cancer subtypes or genotypes for lung cancer treatment. For example, CK2 inhibitors (TBB, TBCA, and hematein) alone and in combination with irradiation have variable effects on cell numbers in four different LCLC and adenocarcinoma cell lines [57]. Quinalizarin also had variable effects in a panel of cell adenocarcinoma lines with or without EGFR mutations. However, an adenocarcinoma cell line with an EGFR L858R + T790M mutation is unaffected by Quinalizarin [55].

CK2 inhibitors alone or in combination with anticancer agents have been successful in decreasing tumor burden in mouse models. Hematein prevents tumor growth in lung adenocarcinoma xenograft models as compared to controls [58]. In vitro, hematein decreases colony formation, phospho-AKT and surviving levels, and increases cleaved PARP [58].

Benavent et al. show that intravenous administration of CIGB-300 (10 mg/kg) in mouse models, markedly decreased lung colonization and metastasis development of murine 3LL cells [59]. Interestingly, after five days of systemic treatment with CIGB-300, tumor cell-driven neovascularization was significantly reduced in comparison to control group. The mechanism could be due to decrease cell adhesion, migration and invasion, MMP-2 and uPA (urokinase plasminogen activator) activity. Altogether their data suggest an important role of CK2 in lung tumor development, suggesting a potential use of CIGB-300 as a novel therapeutic agent against lung cancer. In 2014, Perera et al. studied the synergistic interactions of the anti-casein kinase 2 CIGB-300 peptide and chemotherapeutic agents in lung and cervical preclinical cancer models [60]. They studied agents such as cisplatin (alkylating), paclitaxel (antimitotic), doxorubicin (anti-topoisomerase II) or 5-fluorouracil (DNA/RNA antimetabolite) in cell lines derived from lung and cervical cancer. They observed that paclitaxel and cisplatin exhibited the best synergistic/additive profile when combined with CIGB-300, according to the combination and dose reduction indices. Such therapeutically favorable profiles may be explained by a direct cytotoxic effect and also by the observed cell cycle impairment (arrest in S and G2/M phases) following incubation of tumor cells with selected drug combinations. Paclitaxel displayed the strongest synergism with CIGB-300 in NCI-H125 and SiHa cell lines, with 5 fold less peptide required to show similar anti-proliferative effects. These findings provide a rationale for combining the anti-CK2 CIGB-300 peptide with currently available anticancer agents in the clinical setting and indicate platins and taxanes as compounds with major prospects.

Similarly, there have been studies combining CX-4945 with Erlotinib, an EGFR tyrosine kinase inhibitor used to treat advanced or metastatic non-small cell lung cancer. Bliesath et al. studied combining CX-4945 and Erlotinib, in vitro and in vivo in models of non-small cell lung carcinoma and squamous cell carcinoma, and demonstrated that it inhibited tumor growth via enhanced inhibition of the PI3K-Akt-mTOR pathway [61]. They also observed a decrease in proliferation, an increase in apoptosis, a synergistic killing of cancer cells in vitro, and an improved antitumor efficacy in vivo. Taken together, these data position CK2 as a valid pharmacologic target for drug combinations and support further evaluation of CX-4945 in combination with EGFR targeting agents [61].

There is currently an open clinical trial using CX-4945 in advanced cancers including lung cancer, Castleman’s disease and multiple myeloma (trial designation: NCT00891280).

##### Urothelial Cancer

Urothelial carcinoma is the most common type of bladder cancer and occurs in the urinary tract system (bladder and related organs). The National Cancer Institute (NCI) estimates 76,960 new cases of bladder cancer and 16,390 deaths from the disease in 2016. It is linked to smoking and some chemical exposures. It can be well managed in early stages with surgery and other approaches (e.g., intravesicle BCG (Bacillus Calmette-Guérin immunotherapy). Advanced disease is responsive to chemotherapy but this does not extend survival markedly. The latest in the treatment is FDA approval of Atezolizumab (anti PD-L1 antibody). Atezolizumab showed partial response in at least 14.8 percent of participants and the effect lasted from more than 2.1 to more than 13.8 months at the time of the response analysis. However, we still need more options for those who are not eligible, do not respond to that or develop intolerable toxicity.

Rationale of CK2 inhibition: CK2α could play a role in invasive bladder cancer. First, immunohistochemical analysis shows that CK2α protein is overexpressed in high-grade invasive urothelial carcinomas as well as carcinoma in situ (pT2-4, CIS), but not in low-grade and noninvasive phenotypes (pTa, pT1) [62]. Second, cyclooxygenase 2 (COX2) and phospho-AKT (phospho-AKT), two proteins that may play a role in the transition to an invasive phenotype as they start to be overexpressed in noninvasive phenotypes (pT1), could be upstream of CK2α expression [62]. Indeed, *COX2* knockdown with *COX2*-siRNA decreased expression of CK2α protein and lead to cell cycle arrest at the G1 phase in urothelial carcinoma cell line, UMUC2. In addition, COX2 inhibitor, Meloxicam, suppresses the growth of invasive orthotropic bladder cancer (established by inoculating KU-7 low-grade papillary bladder cancer cells into the urinary bladder of nude mice) correlating with dowregulation of CK2α protein and phospho-AKT level [62]. Third, *CK2α* knockdown with *CK2α*-siRNA fully eliminates uPA activity in UMUC2 cells and reduces uPA activity by half in a human urothelial carcinoma cell line, UMUC6, generated by long-term culture in doxorubicin [62]. uPA has several functions, including the remodeling of extracellular matrix and decreasing cellular adhesion. Fourth, CK2α protein was also increased in the lumen of exosomes secreted from human metastatic transitional cell carcinoma cells in contrast with non-metastatic cells [63]. This is significant because exosomes are proposed to facilitate invasion and metastasis [64]. These data indicate a possible role for CK2α in bladder cancer metastasis. 

CK2α’ and CK2β transcript levels are deregulated in bladder cancer tissues [65], suggesting the possibility that CK2α’ and CK2β proteins are also deregulated in bladder cancer. Determining the levels of these two other proteins during bladder cancer progression will help determine the extent of use of CK2 inhibitors in bladder cancer.

Integration into treatment of bladder cancer: To our knowledge there are no studies on CK2 inhibition in preclinical models of bladder cancer or open clinical trials specifically focused on urothelial cancer. Promising areas for study could include combination of CK2 inhibitors with COX2 inhibitors (see above), immunotherapy or chemotherapy.

##### Head and Neck

Head and neck squamous cell cancers are linked to smoking and human papilloma virus (HPV) (see below). Early stage cancers are well managed with surgery, radiation and chemotherapy. However, advanced or recurrent head and neck cancer is a major problem since chemotherapy provides little survival benefit. A recent study showed benefit of immunotherapy with pembrolizumab (a PD-1 antibody) in advanced head and neck squamous cell cancer. Nonetheless there is a strong need for new therapies in advanced or recurrent head and neck cancer when surgery or radiation are no longer possible.

Rationale of CK2 inhibition: CK2 transcripts, proteins and activity are elevated in head and neck squamous cell carcinoma (HNSCC) tumors and cell lines [37,38,65,66,67,68], and are proposed to be a prognostic marker for HNSCC [65,66,67,68]. The increase in CK2α, CK2α’ and CK2β transcript in HNSCC could be due, at least in part, to DNA amplification [38]. As for CK2 proteins, normal oral mucosa and HNSCC tumor samples show staining of CK2α, CK2α’ and CK2β in cytoplasm and, prominently, in the nucleus [37].

CK2 is essential for HNSCC cell viability, proliferation and cell cycle regulation. Thus, antisense CK2α reduce cell numbers [37,69] and induces apoptosis [37,70] and antisense CK2β induced apoptosis in HNSCC [37,69]. Individual knockdown of *CK2α*, *CK2α’* and *CK2β* arrests HNSCC cells at G0/G1. Similarly, CX-4945 decreases cell numbers, induces cell cycle arrest at S or G2/M, and increases apoptosis in HNSCC cell lines [38].

The differential effects of knockdown of CK2α, CK2α’ and CK2β suggest that these proteins play independent roles in HNSSC. For example, CK2α and CK2β knockdown, but not CK2α’, leads to decreased cell migration of HNSCC cell lines [37]. Individual knockdown of CK2α, CK2α’ and CK2β differentially affect gene expression of key cell proliferation, survival and tumor suppressor genes in HNSCC that are WT for P53, further suggesting that the three genes play independent roles in HNSSC [37]. Similar effects on cell proliferation, survival and tumor suppressor genes are shown using CX-4945 in HNSCC cells WT for P53. However, gene expression changes in mutant P53 cells exposed to CX-4945 were qualitatively different [38].

Several mechanisms for CK2’s action in head and neck cancer are proposed including activation of signaling pathways, EMT processes and cancer stem cell regulation. Thus, HNSCC show elevated levels of NFκB, a factor required for HNSCC cell survival [71]. In HNSCC, NFκB increases could be due to CK2α activity through activation IκB kinase (IKK), a known activator of NF-κB [72]. Indeed, knockdown with siRNA of *CK2α*, CK2α’ and *CK2β* independently decreased NF-κB activity [37]. This suggests the importance of CK2 in HNSCC, and, moreover, the importance of CK2α’ and *CK2β*, both understudied CK2 genes in cancer.

CK2 activity is required for post-translational stabilization of Twist, a transcription factor involved in EMT processes [73]. CK2 also regulates other EMT-linked proteins. For example, CK2 knockdown also decreases protein levels of slug, snail and vimentin, and increases E-cadherin levels in laryngeal carcinoma cells [74]. CK2 is also required for IL-6 dependent cell migration of HNSCC cells [73]. This is significant because IL-6, a cytokine, is upregulated in HNSCC correlating with recurrence and low patient survival [75,76,77]. These data indicate a role for CK2 in the metastatic potential of head and neck cancers.

CK2 activity is required for cancer stem cell-like cell maintenance, tumor sphere formation and proliferation, and for the expression of cancer stem cell genes and proteins (e.g., Nanog, Oct4, and Sox2) in HNSCC [78]. Cancer stem cell genes are important in tumor initiation and therapeutic resistance, positing a role for CK2 in tumorigenesis and therapy resistance in HNSCC.

Integration into clinical care of head and neck cancer: Preclinical models show that CK2 inhibitors reduce tumor burden in head and neck cancer. HNSCC xenograft tumor models (tongue, hypopharyngeal and laryngeal carcinomas) show that treatment with CK2 inhibitors (RNAi-CK2α/α’containing nanocapsules) can significantly reduce tumor volume, reduce number of metastasis and increase the survival of mice [79]. In addition, tumors from CK2α/α’-RNAi-treated mice show reduced staining of pro-proliferative proteins (e.g., Cyclin D1) and increased levels of tumor suppressors (e.g., P53) compared to tumors from control mice [37]. Nanoencapsulated CK2α/α’ RNAi did not affect mouse body weight [79]. In contrast, CX-4945 had no effect on the survival of mice and minimal effect on tumor volume in a HNSCC xenograft tumor model, and did not synergize in a combination with a MEK inhibitor (PD-901). This lack of effect of CX-4945 in vivo could be due to an ineffective drug dose scheme, lack of intake in tumor cells, or the particular cell line genotype.

The likely first use in clinical trials will be in advanced head and neck cancer given the limited treatment options for this type of tumors. To our knowledge there are no active trials of CK2 inhibitors directed against head and neck specifically. CK2 inhibitors could be tested out as single agents in refractory disease or combined with chemotherapy or immunotherapy. In cases where the cancer is linked to HPV, there may be an additional rationale for use of CK2 inhibitors (see discussion below of HPV related cancers).

##### Mesothelioma

Malignant mesothelioma is a rare neoplasm linked to asbestos exposure. It has an extremely poor prognosis with median survival of 4 to 13 months for untreated patients and 6 to 18 months for treated patients, regardless of therapeutic approach. Combination chemotherapy using cisplatin and pemetrexed is the standard regimen for patients with unresectable disease. For selected minority of patients, a monoclonal antibody, bevacizumab has shown some promise but still awaiting regulatory approval.

Rationale of CK2 inhibition: CK2α transcript is deregulated in mesothelioma tissues and cell lines [65,80]. CK2α staining is elevated in mesothelioma tissue sections and cell lines [80]. Moreover, the *CK2α* pseudogene (*CK2αP)* could be a prognostic factor in mesothelioma [65].

CK2 plays a role in the control of Hedgehog (Hh)/Gli1 signaling, a pathway that is aberrantly upregulated in mesothelioma [81,82]. Thus, CK2α upregulation in mesothelioma correlates with upregulation of the transcript and protein for Gli1, the transcription factor of Hh signaling. CK2α activity is sufficient and necessary (CK2α-siRNA, CX-4945) to activate a Gli1 reporter in mesothelioma cell lines [80]. Furthermore, CX-4945 treatment led to a decrease in mesothelioma cell proliferation [80]. These indicate that CK2 inhibition is a potential treatment for mesothelioma potentially through the regulation of Hh signaling.

Integration into clinical care of mesothelioma—To our knowledge there are no active trials of CK2 inhibitors in mesothelioma. However, this is certainly a disease in need of new therapies, especially in the advanced stages. Potential approaches would include use of CX-4945 or CIGB 300 in combination with cisplatin or pemetrexed.

#### 2.1.2. Gastrointestinal Cancers (Often Related to Chronic Inflammation)

##### Hepatocellular Cancer

The major etiological factors for hepatocellular cancer include chronic infection with hepatitis B or C, alcohol cirrhosis, or steatohepatitis. There are limited options for treatment for hepatocellular cancer when not amenable to transplant or surgery. Local ablative therapies or tyrosine kinase inhibitors are used often and these measures improve survival by several months in general. Immunotherapy is also being studied for advanced hepatocellular cancer. Clearly there is need for additional therapies.

Rationale of CK2 inhibition: Hepatocellular cancer (HCC) samples show high expression of CK2 gene transcripts, proteins and activity [6,65,83,84,85]. Levels of CK2 transcripts and proteins are proposed as prognostic markers for HCC [6,65,83]. Interestingly, a variant of CK2α, CK2α’’, is highly expressed in the liver and is required for membrane protein trafficking and has limited nuclear localization [86]. The role of CK2α’’ in HCC has not been studied.

In mouse xenograft models, injection of hepatoma cells overexpressing CK2α leads to higher tumor volume than injection of the corresponding untransfected hepatoma cells, further indicating the oncogenic nature of CK2α [6]. In vitro, CK2α overexpression increases proliferation, colony formation, migration and invasion, and inhibits apoptosis in HCC cell lines [6]. This suggests that CK2 may be involved in the control of diverse cellular processes during HCC progression.

Conversely, *CK2α* knockdown decreases proliferation and colony formation, causes G2/M arrest, and increases apoptosis in HCC cell lines [6,83,85]. *CK2α* knockdown also inhibits migration and invasion in HCC cell lines [6,84]. Several mechanisms have been proposed for these effects of *CK2α* knockdown in HCC cell lines. Among the mechanisms proposed are downregulation of MMP and EMT-linked proteins (snail, slug, vimentin) and increasing E-cadherin expression [84]; inhibition of Hedgehog signaling [84]; and downregulation of phospho-Akt, apoptotic-marker genes and p53 [6]. Additionally, CK2 inhibition can increase Natural Killer (NK) cell-mediated apoptosis of liver cancer cells suggesting that CK2 could increase tumor immunity [87]. Similarly, chemical inhibition of CK2 in HCC cells leads to decreased proliferation (e.g., DMAT [85]) and increased apoptosis (e.g., emodin, [87]).

Integration into clinical care of HCC: Preclinical studies show that CK2 inhibitors reduce tumor burden alone and in combination with other agents. Thus, in mouse xenograft models of HCC, DMAT and shCK2α inhibit tumor growth [6,85]. DMAT acts by decreasing proliferation but not cell survival or angiogenesis and, importantly, did not induce liver damage [85]. DMAT’s effects may be mediated by reducing NFκB and Wnt/β-catenin signaling activation [85]. Moreover, CK2 inhibitors increase the effectiveness of chemotherapeutics on HCC proliferation (5-fluorouracil (5-FU), doxorubicin or sorafenib) [85,87]. These suggest that CK2 inhibitors could be used as potential therapeutics for liver cancers, alone or in combination in other drugs.

##### Gastric Cancer

Gastric adenocarcinoma is a major cause of cancer death worldwide, and its development is linked to *Helicobacter pylori* infection and chronic atrophic gastritis. There are several lines of chemotherapy available to extend survival in advanced disease. Anti-HER 2 antibodies are also useful in subset of patients, and anti VEGF receptor antibodies are a part of second line therapy. Additional therapies are clearly needed since survival in advanced gastric cancer is limited to approximately 1 year even when using all available treatments.

Rationale of CK2 inhibition: CK2 transcripts and proteins are elevated in gastric cancer [88], and CK2 is proposed as a prognostic marker for gastric cancer [65,88,89]. Interestingly, CK2 activity is upregulated after *H. pylori* infection of gastric epithelial cells [90]. The upregulation of CK2 activity is not due to increased CK2α protein levels, however, CK2α’ and CK2β expression are still to be studied in these tumors [90].

CK2 inhibition leads to decreased cell proliferation and migration of gastric cancer cells by decreasing MMP expression [88]. CK2 inhibition also leads to apoptosis in human gastric carcinoma cells [91]. 

Importantly, CK2 is associated with *H. pylori*-infection in gastric cells. In gastric epithelial cells, CK2 is necessary for *H. pylori*-induced cell migration and invasion [90]. The underlying mechanism is the phosphorylation of α-catenin by CK2 that results in the depletion of α-catenin at the membrane and subsequently, the disruption of the α-catenin/β-catenin complexes at the membrane. Indeed in *H. pylori-*infected patient tissue sections, membrane α-catenin, β-catenin and E-cadherin levels are decreased compared with non-infected tissue samples [90]. The disruption of these membrane complexes leads to β-catenin accumulation in the nucleus of *H. pylori*–infected tissue sections. This is important because, nuclear accumulation of β-catenin is a requisite for activation of Wnt/β-catenin signaling. Indeed, in gastric epithelial cells, *H. pylori*-infection leads to increased Wnt/β-catenin reporter activity and Wnt target gene MMP7, a secreted protein that breaks down the extracellular matrix, in gastric cells [90]. Additionally, CK2 could be associated to gastric carcinoma also through phosphorylation of DBC1 (deleted in breast cancer 1) [88].

Integration into clinical care of gastric cancer: CK2 may play a role in resistance to chemotherapy. Thus, CK2α protein levels are elevated in cisplatin resistant gastric cancer cells. Moreover, CK2 has been proposed to help mediate cisplatin resistance in gastric cancer cells [92].

There are no active trials of CK2 inhibitors focused specifically on gastric cancer. However, there is a strong need for novel therapies for this common cancer. The likely area for initial trials of CK2 inhibitors will be in advanced disease after progression on the first two approved lines of therapy.

##### Esophageal Cancer

There are two major types of esophageal cancer: adenocarcinoma and squamous cell carcinoma. The main epidemiological link for adenocarcinoma is chronic reflux esophagitis, and the incidence of this cancer is increasing. Squamous cell carcinoma is mainly linked to alcohol and tobacco use, and its incidence is declining. Localized disease is curable in some cases with combinations of chemotherapy, radiation and surgery but there are limited options for treatment of advanced or recurrent disease.

Rationale of CK2 inhibition: To our knowledge there is no data on expression of CK2 proteins in esophageal cancer. However, CK2 gene transcripts are deregulated in esophageal cancer [65]. Importantly, esophageal cancer cell lines show differences in CK2 activity levels despite similar levels of CK2α expression [93]. This suggests that expression of the other CK2 proteins may be deregulated, although other mechanisms are possible. CK2 activity is also found elevated in TRAIL (TNF-related apoptosis-inducing ligand)-resistant esophageal cancer cell lines compared to non-resistant cell lines [94]. This suggests that CK2 may play a role in resistance to therapy in esophageal cancer.

CK2 has been linked to invasive phenotypes in esophageal cancer cells. Thus, *CK2α* overexpression increases invasiveness in esophageal cancer cells through the nuclear receptor corepressor (NCoR) [93]. NCoR represses transcription of the chemokine IFNγ-inducible protein-10 (IP-10), an antitumoral gene that inhibits tumor proliferation and metastasis [93]. The ability of CK2α to promote invasion is also linked to EMT-linked genes. Thus, CK2α overexpression results in decreased E-cadherin expression and increased N-cadherin and vimentin expression [93,95]. These changes in EMT-linked proteins lead to resistance to anoikis, a type of induced programmed cell death when anchorage-dependent cells detach from the surrounding extracellular matrix [95]. This suggests that CK2 could act to promote metastasis in esophageal cancer.

CK2 may be mediating inhibition of 5-fluorouracil (5-Fu)-induced apoptosis by IGF-1 in esophageal carcinoma cells [96]. Since 5-FU is a key ingredient in treatment of esophageal cancers (and other gastrointestinal tract cancers), this suggests that combining CK2 inhibition with 5-FU may be an effective strategy. 

Integration into clinical care of esophageal cancer: There are no active trials of CK2 inhibitors focused specifically on esophageal cancers. The most likely initial use is in advanced or recurrent cancer, where survival is poor and chemotherapy is of limited effectiveness.

##### Cholangiocarcinoma

Cholangiocarcinomas are rare malignancies arising from the epithelial cells of the intrahepatic and extrahepatic bile ducts. The etiology is not clear, but there is a linkage to chronic liver fluke infection, chronic hepatitis C infection and possibly to Hepatitis B and HIV. The first line chemotherapy regimen in advanced disease includes gemcitabine plus cisplatin, which yields an approximate 4-month survival benefit compared to gemcitabine alone. Overall cures are rare when the cancer is not resectable and there is a strong need for new agents, especially in advanced or recurrent disease.

Rationale of CK2 inhibition: CK2 has been linked to cholangiocarcinoma (CCA) tumorigenesis. CK2β staining is higher in CCA tumor sections compared to normal tissue liver sections [97]. CK2β staining is found in the cytoplasm and more prominently in the nucleus. In addition, high CK2β staining is associated with higher tumor stage, higher histological grade and high serum CEA (carcinoembryonic antigen) level. These suggest a role for CK2 in CCA progression and invasion. High CK2β staining correlates with lower patient survival, and it is an independent prognostic factor in CCA [97]. In addition, CK2α was specifically detected in plasma of CCA patients suggesting that CK2α is a potential biomarker for CCA diagnosis [98].

It has been proposed that XIAP (X-Linked inhibitor of apoptosis protein) could be downstream of CK2β upregulation in CCA, as CK2 activity is required for XIAP levels [99]. This is important because XIAP is upregulated in CAA, and high levels of XIAP correlate with low patient survival [97].

Integration into clinical care of cholangiocarcinoma: Currently, CX-4945 is being tested in phase I/II study in combination with gemcitabine and cisplatin in patients with cholangiocarcinoma. In this context, CX-4945 may inhibit DNA repair, and be particularly effective when combined with agents such as cisplatin that cause DNA damage. The objective of this trial is to determine the maximum tolerated dose (MTD), followed by a randomized study that compares antitumor activity in cholangiocarcinoma patients receiving the standard of care gemcitabine plus cisplatin versus CX-4945 at the combination MTD with gemcitabine plus cisplatin. This is a multicenter study in the United States, South Korea and Taiwan with estimated study completion date of December 2017(NCT02108282).

##### Colorectal Cancer

The most common type of colorectal cancer is adenocarcinoma (95%). The precursor to adenocarcinoma is adenomatous polyps (adenomas), which are a precancerous condition. Treatment includes surgery, chemotherapy, biological therapy, and radiation therapy.

Rationale of CK2 inhibition: CK2 gene transcripts are elevated in colorectal cancer [19,89]. Notably, CK2α protein is overexpressed in the nucleus of cells in tumor tissue sections, compared to normal colorectal and adenoma tissue sections [100]. Elevated levels of nuclear CK2α correlate with poor prognosis in patients with colorectal cancer, with increased invasion depth, node involvement, American Joint Committee on Cancer (AJCC) stage, poorer differentiation and overall decreased survival rate [101].

Inhibition of CK2α, by siRNA or CK2α inhibitor emodin, led to inhibition of colorectal carcinoma cell proliferation, G0/G1 cell cycle phase arrest, inhibition of cell division, increase in p53/p21 expression, downregulation of c-myc, and decreased cell motility and invasion [100].

CK2 has been shown to regulate Wnt/β-catenin signaling [5]. Importantly, Wnt/β-catenin signaling is major player in colorectal cancer, as it is proposed to be the first hit to be deregulated in colon cancer progression [102]. CK2 also promotes colon cancer cell invasion by increasing stability of a membrane metalloprotease, endothelin-converting enzyme-1c (ECE-1c) [103]. ECE-1c is involved in endothelin-1 synthesis, which also participates in invasion in vivo in breast, ovary, and prostate cancer cells [104,105,106,107]. CK2 phosphorylates ECE-1c at the N-terminal end, stabilizing it. Expression of full-length ECE-1c mutants mimicking CK2 phosphorylation led to increased invasion of colon cancer cells. Conversely, expression of full-length ECE-1c mutants that cannot be phosphorylated by CK2 led to decreased invasion of colon cancer cells. Together these demonstrated the relation of CK2 and ECE-1c protein stabilization by phosphorylation in increasing invasion of colon cancer cells, demonstrating a mechanism by which CK2 can promote invasion of colon cancer.

Integration of CK2 inhibition into therapy of colorectal cancer: There are no active trials of CK2 inhibitors focused specifically on colon cancer. In vitro data suggest that CK2 may play a role in resistance to chemotherapy. Thus, CK2 protects against colon carcinoma cell apoptosis induced by TRAIL (TNF-related apoptosis-inducing ligand) [108]. Indeed, CK2 inhibition with DRB leads to increased TRAIL-induced apoptosis in colon carcinoma cells, correlating with increased TRAIL-induced death-inducing signaling complex (DISC) formation, caspase-8 cleavage, and Bid cleavage, leading to proapoptotic factor release from the mitochondria. *CK2α* knockdown with *CK2α* shRNA also increased TRAIL sensitivity in human colorectal adenocarcinoma cells. These suggest a potential for CK2 inhibition in overcoming tumor resistance to therapy.

##### Pancreatic Cancer

Pancreatic adenocarcinoma remains a common and still highly lethal malignancy. Most cases are not amenable to curative surgery and there is no screening modality to detect this cancer in the general population at risk. Treatments for advanced disease have limited survival impact, so new therapies are desperately needed.

Rationale for CK2 inhibition: *CK2α* and *CK2α’* knockdown, DRB, and apigenin induce apoptosis of pancreatic cancer cells [109,110,111]. Some of these effects can be mediated by a reduction of NF-κB-dependent transcriptional activity [109]. In addition, D11, a potential inhibitor of CK2, reduced phosphorylation of two biomarkers for CK2 activity, CDC37 and PTEN [110].

There may be an important role for CK2α’ in pancreatic cancer. Thus, knockdown of *CK2α’* was more efficient in inducing apoptosis than *CK2α* knockdown in PANC-1 cells, a gemcitabine resistant cell line. *CK2α* or *CK2α’* knockdown sensitizes cells to gemcitabine, correlating with increased Jun-amino-terminal kinase (JNK) phosphorylation and phospho-p70S6K (T389), respectively. Authors speculate that CK2α may be sequester in an inactive from in oligomers of the tetrameric CK2α2β2 while, CK2α’2β2 tetramers, that cannot oligomerize, will be active in these cells [112].

Integration of CK2 inhibition into therapy of pancreatic cancer: Preclinical studies show that CK2 inhibitors reduce tumor burden, alone or in combination with other inhibitors. Thus, in a mouse xenograft model of pancreatic cancer, CX-4945 inhibits tumor growth and decreases p21 staining [44]. Moreover, in an orthotopic mouse xenograft model of pancreatic cancer, intraperitoneal injection of *O*-methyl modified CK2α siRNA together with a transfection reagent results in a trend towards decreased tumor volume, and a significant increase in apoptosis [111]. A larger and significant decrease in tumor volume is found when CK2α siRNA is used in combination with PAK7 and/or MAP3K7 siRNAs [111]. None of these treatments affect mouse weight. These suggest that CK2 inhibitors could be used as potential therapeutics for pancreatic cancers.

#### 2.1.3. HPV-Related Cancers: Cervical, Head and Neck, Anal and Penile

Cancers related to HPV infection are highly prevalent worldwide and do not respond well to treatment in advanced stages. Cervical cancer and anal cancer are particularly prevalent and problematic in patients with HIV infection and AIDS. There is evidence that CK2 plays a specific role in the mechanisms through which HPV induces cancer, and hence CK2 inhibition has received special attention in these cancers.

Rationale for use of CK2 inhibition in cervical cancer: CK2 gene transcripts are deregulated in cervical cancer, and the *CK2α* pseudogene, *CK2αP*, could be a prognostic marker in cervical cancer [65]. To our knowledge there are no studies showing expression of CK2 proteins in cervical cancer, nonetheless, CK2 activity is higher in HPV-immortalized cell lines compared to normal keratinocytes [113]. Conversely, CK2 regulates HPV through the HPV-16 E7 viral oncoprotein [113,114]. 

CK2 inhibitors affect cervical cancer cells. Thus, CIGB-300 inhibits cervical cancer cell line proliferation [60]. Moreover, CIGB-300 synergized with paclitaxel and doxorubicin, and was additive in combination with cisplatin (CDDP) [60]. CK2 could also play a role in cervical stem cancer cell maintenance, as apigenin inhibits HeLa tumorsphere formation and self-renewal capacity while, conversely, CK2α overexpression increases self-renewal capacity [115]. The tumorsphere assay is an analysis of the self-renewal capability of cancer stem cells, which is a population with tumor initiation, drug resistance, and metastasis properties.

Integration into clinical care of cervical cancer and by extension other HPV related cancers: CK2 could be a potential therapeutic target in cervical cancer. The efficacy of CK2 inhibitor in cervical tumor remission was first tested on mouse xenograft models. CIGB-300 diminished tumor growth, even after cessation of treatment [43,60]. In addition, CIGB-300 plus cisplatin significantly reduced tumor growth and increased mouse survival in a cervical mouse xenograft model [60].

Moreover, the first human clinical trial of the CK2 inhibitor CIGB-300 demonstrated potential clinical benefit for cervical cancer [116]. In this study, thirty-one women with cervical cancer were administered CIGB-300 intralesionally at four increasing doses over five consecutive days. Side effects were minimal even with the highest dose, and 75% of patients had significant lesion reduction. Strikingly, 19% of patients had full histological regression and 48% of patients who were previously HPV DNA-positive were negative at the end of the trial [116]. At the one-year follow up, there were no recurrences or adverse events. Interestingly, four patients conceived, of which two were infertile prior to the intervention. Pharmacokinetic studies with CIGB-300 have established a treatment plan for phase II trials [117]. Therefore CK2 inhibition is tolerable in humans, and is a promising treatment for cervical cancers, alone or in combination with chemotherapeutic agents [60].

There is currently an open clinical trial in Argentina, where investigators are using CIGB-300 in patients with squamous cell carcinoma or adenocarcinoma of the cervix stage IIA and IIB FIGO classification in a concurrent fashion with external radiotherapy, endocavitary brachytherapy and weekly systemic cisplatin (trial designation: NCT01639625)

#### 2.1.4. Other Solid Tumors: Glioblastoma, Melanoma, Ovarian Cancer, Prostate Cancer, Breast Cancer and Renal Cell Carcinoma

##### Glioblastoma

Glioblastoma (GBM) is a highly lethal cancer and there are limited treatment options once it recurs after surgery and radiation (combined with chemotherapy). Cure rates for this cancer are low and there is a strong need for new therapies.

Rationale of CK2 inhibition: Numerous reports have recently shown a role for CK2 in GBM tumorigenesis. First, CK2 transcripts and proteins are overexpressed in GBM samples. Thus, CK2α transcript and protein levels and staining are elevated in GBM tumor samples [21,65,118,119,120]. The increase in CK2α transcripts may be due to changes in gene dosage. *CK2α* had gene dosage gains in more than fifty percent of human GBM cases [21]. CK2α’ gene dosage gains have also been reported in GBM [21]. Expression of CK2α transcripts correlates with lower survival [119], although this may be controversial [65]. In addition, CK2α but not CK2β is overexpressed in tumors from a preclinical model of GBM (GL261 cells) compared to normal tissue. The authors suggest that the unbalance in the ratio CK2α/CK2β in GL261 tumors should be considered in preclinical studies [121]. 

Second, CK2α or CK2β overexpression is sufficient to increase GBM cell proliferation and colony formation in GBM cell lines [119]. 

Third, CK2 is necessary in GBM for cellular processes and signaling pathway activation. Thus, CK2 inhibition (CX-4945, siRNA, RNAi, TBB, DMAT, DRB, apigenin, etc.) in GBM cell lines leads to increased apoptosis, S or G2/M cell cycle phase arrest, and decreased adhesion, migration and colony formation [20,21,118,119,122,123,124]. The downstream mechanism by which CK2 inhibition causes these effects in GBMs is still being studied. Among the proposed mechanisms are the downregulation of the Wnt/β-catenin, JAK/STAT-3, NF-κB and PI3K/Akt pathways, and enhanced DNA-PK activity and autophagy [20,21,119,120,125]. 

CK2 inhibition sensitized GBM cells to TNFα-induced apoptosis, through mechanisms involving NFκB, p53 and SIRT1 [118]. This is relevant because GBM cells are often resistant to TNFα-induced apoptosis mediated through NF-κB activation [118]. Recently a phospho-proteomic study indicated that a constitutively active epidermal growth factor receptor (EGFRvIII) overexpressed in GBM may regulate the activity of CK2α in GBM [126]. 

In addition, CK2α is necessary for expression of Oct4 and Nanog, two genes involved in glioblastoma-initiating cell proliferation, in cells and tumor spheres from GBM patient cell lines [119]. These data suggests that CK2 activity is required to maintain stem cell phenotypes and self-renewal in GBM [119]. Therefore, CK2 could be a potential target for the treatment of GBM.

Integration of CK2 inhibition into treatment of glioblastoma: Preclinical GBM xenograft models show that several CK2 inhibitors are effective in inhibiting glioblastoma tumor growth and increasing the survival of mice [21,119,123,124,127]. In addition, *CK2* knockdown alone or in combination with *EGFR* knockdown increased mouse survival and necrosis in the tumor tissue of GBM xenograft mouse models [127]. CK2 inhibition in xenograft GBM tumors lead to decreased activation of STAT-3, NF-κB, c-myc and AKT, and decreased EGFR expression, suggesting that CK2 controls several pro-survival and pro-proliferative signals in vivo [21,127]. Hence, CK2 inhibitors may have a role in preventing recurrence after surgical resection along with radiotherapy with concurrent and adjuvant Temozolomide.

##### Melanoma

Therapy for advanced melanoma has been improving with use of signal transduction inhibitors for tumors expressing BRAF mutations. The other recent major advance in therapy of advanced melanoma or melanoma at high risk of recurrence after surgery is the use of immune checkpoint inhibitor antibodies. However, melanoma is still an aggressive and often lethal disease and its incidence is increasing.

Rationale of CK2 inhibition: *CK2α* transcript is upregulated in 15% of melanoma tumor samples [128]. To our knowledge there is no data on expression of CK2 proteins in melanoma. However, CK2 activity is elevated in metastatic melanoma samples compared to dermal nevus [129], suggesting that there is aberrant expression of CK2 transcripts and/or proteins in melanoma. Strengthening this notion, protein levels of CK2α are increased in melanoma cell lines [128].

CK2 inhibition with 7,7’-Diazaindirubin diminishes proliferation in melanoma cell lines [130]. Importantly, CK2 has been linked to sensitivity to BRAF inhibitors. First, CK2α overexpression in BRAF-mutant melanoma cells decreased sensitivity to BRAF inhibitors (vemurafenib, dabrafenib) and MEK inhibitor (trametinib) [128]. Conversely, *CK2α* knockdown sensitized BRAF-mutant melanoma cells to vemurafenib. Interestingly, combination of CK2 inhibitor CX-4945 and BRAF inhibitor vemurafenib additively inhibited proliferation in BRAF mutant patient-derived melanoma cell lines [131]. This is significant because patients with BRAF mutations become resistant to vemurafenib, and the novel therapies being explored aim to target several signaling pathways. One interesting finding is that the effect of CK2α overexpression in melanoma is not dependent on its catalytic activity [128]. 

Integration of CK2 inhibition into clinical care of melanoma: It is still true that most patients with metastatic melanoma succumb to their disease, therefore trials in advanced melanoma either as single agents or in combination with signal transduction inhibitors or immunotherapy will be of great interest.

##### Ovarian Cancer

Ovarian cancer is hard to detect in early stages and difficult to cure with current modalities in advanced stages.

Rationale for CK2 inhibition in ovarian cancer: CK2α transcript expression is elevated in 518 serous cystadenocarcinomas samples as compared to 8 fallopian tube sample controls [132]. In a culture model of epithelial ovarian tumorigenesis, CK2α protein expression is highest in a metastatic cell line compared to a neoplastic and tumorigenic cell line, and to the control (normal and pre-neoplastic, non-tumorigenic) cell lines [133], suggesting a role for CK2 in ovarian cancer progression.

CK2 may play a role in the maintenance of cancer stem cells, as CK2α protein levels are higher in the tumorspheres from SKOV3 cells that are contain only cancer-stem cells, compared with the parental cells that are a mix of cancer stem and non-stem cells. In addition, CK2α overexpression increases the sphere formation of SKOV3 cells over two fold, and inhibition of CK2 (apigenin and CK2α siRNA) decrease sphere forming efficiency. These two effects correlate with increased and decreased expression of *Gli1*, which is amplified in ovarian cancer stem-like cells respectively [134].

Integration of CK2 inhibitors into treatment of ovarian cancer: Combination therapies with CK2 inhibitors have studied in cell cultures and xenograft models. In cell cultures, CX-4945 synergizes with cisplatin and gemcitabine to increase apoptosis in p53 WT (A2780) but not in p53 null (SKOV-3) cells, and to increase mitotic catastrophe in SKOV-3 cells [135].

CX-4945 in combination with dasatinib, a tyrosine kinase inhibitor of the Src-family kinases, promote apoptosis in a panel of human epithelial ovarian carcinoma cell lines. Cell lines with low *CSNK2A1* transcript expression had lower viability in the presence of dasatinib, therefore *CSNK2A1* transcript levels are proposed to be a predictor for dasatinib sensitivity. Indeed, a 3:1 ratio of CX-4945:Dasatinib were better in cell lines with low *CSNK2A1* transcript expression, while a 8:1 or 20:1 ratio was synergistic for cell lines with high transcript levels of *CSNK2A1*. Authors suggest that CSNK2A1 levels should be analyzed before treatment with dasatinib [132].

In a mouse xenograft model of non-high grade ovarian cancer (IGROV-1 cells), CX-4945 treatment prevented tumor growth, decreased vascular area and proliferative index, and prevented mRNA expression of TNF, IL-6, and VEGF. CK2 inhibition reduces the release of these factors in IGROV-1 and SKOV3ip1 cells, and in primary ovarian cancer cells from ascites [136]. 

In a xenograft model of high-grade ovarian cancer (A2780 cells), a combination of CX-4945 with three drugs (cisplatin, Carboplatin, and gemcitabine) extended the time-to-endpoint two fold as compared to the untreated mice. Specifically, carboplatin synergizes with CX-4945 while cisplatin and gemcitabine had an additive effect on tumor growth inhibition However, the combination of cisplatin and CX-4945 resulted in mouse weight loss [135]. Hence, CK2 inhibitors could be used for the treatment of advanced ovarian cancer.

##### Prostate Cancer

Prostate cancer is the second most common cancer in men (after lung cancer) and remains lethal in advanced stages despite recent advances in therapy.

Rationale for CK2 inhibition in prostate cancer: CK2 protein levels and activity are deregulated in prostate cancer and prostate cancer-derived cells. Human benign prostatic hyperplasia (BPH; *n* = 31) and prostate cancer (*n* = 30) tissue sections had higher staining for CK2α and NF-κB compared to normal prostate tissue specimens [137]. Furthermore, CK2α staining in sections is higher in malignant compared to normal human prostate glandular cells. Total CK2α immunostaining correlated with poorly differentiated tumors (high Gleason scores) and locally aggressive tumors (high cT). In both normal and tumoral glands, there is higher staining in the nucleus than in the cytoplasm. However, nuclear, but not cytoplasmic, staining of CK2α in prostate cancers correlates with high cT stages, higher Gleason scores, and more potential capsular involvement (lymphatic or perineural invasion). All these are established prognostic factors for prostate cancer, therefore CK2α nuclear staining can be a prognostic marker [138].

In contrast, decreased CK2α and CK2α’ protein levels are observed in xenograft tumors relative to cultured cells from cell lines including C4-2 cells, a metastatic subline of LNCaP (androgen-sensitive human prostate adenocarcinoma cells) and PC3-LN4 cells, a lymph node derived cell line from repeated orthotopic injections of PC-3 cells (bone-metastatic derived prostate adenocarcinoma cells). Intriguingly, the transcript levels in the xenograft tumors doubled compared to their respective cultured cell lines. Authors speculate that this decrease in CK2α/α’ protein levels in xenograft tumors is due to the presence of mouse stromal cells within the lysate. Intriguingly, CK2α’ protein levels were greater in BPH-1 cells compared to prostate cancer cell lines [139]. It is possible that CK2α’ overexpression explains the intense staining in human BPH tissue sections compared to controls found by other authors [140]. As it is found in other tumor types, despite having the same amount of CK2α protein and mRNA levels, some prostate cell lines show different CK2 activity. For example, PC-3 (bone-metastatic derived prostate adenocarcinoma) cells have 8 times more CK2 activity than RWPEI (normal prostate) cells and 3 times more activity than LNCaP cells (androgen-sensitive human prostate adenocarcinoma cells) [141]. Importantly, CK2 activity levels correlate with increased of invasion potential (matrigel invasion assay) in these cell lines [141].

CK2 inhibition (DMAT, TF, TBB, TBCA, siRNA, apigenin, and KI-CK2α) reduces cell proliferation in prostate cancer cell lines [49,142,143,144,145,146], and TF, CX-4945, DMAT, TBB, TBCA, and apigenin increase apoptosis [49,142,146,147,148,149,150]. CX-4945, apigenin, and TBCA induced cell cycle arrest in G2/M phase [146,148]. Interestingly, only when DMAT and CK2α/α’ siRNA are nanoencapsulated, they specifically decreases proliferation in prostate cancer cell lines (PC3-LN4), but not benign cell lines (BPH-1) [144]. In addition to PC3-LN4, nanoencapsulated CK2α/α’ siRNA also affects prostate cancer cell line C4-2, but not normal prostate epithelial cell lines (PrEC) [144].

A number of mechanisms are proposed to underlie the role of CK2 in prostate cancer. For example, apigenin, TBCA, and CK2α or CK2α’ siRNA decrease nuclear translocation of AR (androgen-receptor) and AR-mediated gene expression in response to an AR agonist, R1881, treatment [146]. TBB sensitizes cells to TRAIL (tumor-necrosis factor-related ligand)/induced apoptosis and glycolysis inhibitors (2-DG) in PC-3 and ALVA-41 cells in a synergistic manner [150,151]. Conversely, overexpression of CK2α partially blocks TRAIL-induced apoptosis, caspase activity and caspase protein levels in ALVA-41 and PC-3 cells [152]. In addition, reactive oxygen species were detected after 6 h of DMAT but not with TBB treatment. After 24 h of TBB or DMAT treatment, γH2AX levels increase in LNCaP cells [142].

Integration of CK2 inhibition into clinical care of prostate cancer: Xenograft models show that CK2 inhibition can decrease tumor burden in mice. Nanoencapsulated-CK2α/α’ siRNA and RNAi [139], and CX-4945 [148] decreased tumor volumes in metastatic PC-3 derived-xenograft tumors. Nanoencapsulated-DMAT, decrease proliferation and CK2α and CK2α’ proteins levels in PC3-LN 4 cells derived-xenograft tumors [143]. 

To date there are no reported human trials of CK2 inhibitors in prostate cancer, but such trials would be reasonable in patients with advanced disease that have progressed on other approved lines of therapy (which include various anti-androgen therapies and chemotherapy). In addition, CK2α nuclear staining may be tried as a prognostic factor in patients with early disease to tailor how closely they should be monitored.

##### Breast Cancer

Breast cancer treatment is now very complex and in many ways highly successful, especially in early stage disease. There are many agents available for treatment of advanced, metastatic breast cancer and molecular typing is key in deciding on the appropriate therapy. Estrogen receptor positive (ER+) breast cancer is largely approached with anti-estrogen therapies which can result in years of disease control even in metastatic disease. Many patients with metastatic, ER+ breast cancer eventually develop resistance to anti-estrogenic approaches, representing a challenge for treatment. HER2 positive (HER2+) breast cancers can be targeted with a range of agents directed against this receptor. Finally, patients lacking receptors for estrogen, progesterone and also lacking over-expression of HER2 (triple negative breast cancer) have currently fever options for therapy apart from chemotherapy and, more recently, PARP inhibitors for some patients. Hence, there are several areas of need for new therapy in advanced or metastatic breast cancer as nearly all such patients eventually succumb to their disease. 

Rationale for CK2 inhibition: There is strong evidence for a role for CK2 in the pathophysiology of breast cancer as CK2 is overexpressed or mutated in breast cancer. In general, human breast tumors show high levels of *CK2α* and *CK2β* and low levels of *CK2α’* transcripts [19,153,154,155]. *CK2α* and *CK2β* transcripts were higher in basal tumors while *CK2α’* was lower in luminal A and B and *HER2* tumors [155]. These aberrations in *CK2* gene transcript expression correlate with changes in copy number variation [153]. In particular, basal tumors had higher gain on *CK2β* and Luminal A tumors show higher loss on *CK2α’* [153,155]. High levels of *CK2α* and *CK2β* transcripts predict lower survival rates [19]. In addition, high *CK2α* transcript expression correlates with increased risk of relapse among breast cancer patients with ERα+ grade 1 or 2 tumors and those receiving hormonal therapy [156]. Moreover, *CK2α* is part of an “invasiveness” gene signature associated with overall survival and metastasis-free survival in breast cancer patients [157].

CK2α activity and protein are elevated in human breast tumors [158,159]. CK2α staining is increased prominently in the nucleus and also in the cytoplasm in breast tumor sections [154,159,160,161]. Importantly, high CK2α staining is an independent prognostic indicator of patient survival and relapse-free survival [154]. High CK2α staining is also associated with distant metastatic relapse and *HER2* expression, and negatively correlated with progesterone receptor (*PR*) expression [154]. 

Similar alterations in expression can be found in breast cancer cell lines. CK2α, CK2α’ and CK2β proteins are expressed in variable levels in breast cancer cell lines [44,153,155]. CK2 transcript and protein expression levels do not correlate in a number of cell lines suggesting that post-transcriptional mechanisms regulate CK2 expression [155]. Similar to tumors samples, breast cancer cell lines (MDA-MB-231 and MCF7) show loss of heterozygosity on *CK2α’*, however, CK2α’ protein was still expressed at high levels [153]. Immunostaining show CK2α and CK2α’ located in the nuclei and cytoplasm in the non-transformed immortalized triple-negative breast cell line MCF10A [155]. 

Elevated levels on CK2α could play an important role in breast cancer, since elevated levels of CK2α have been show to be oncogenic in mouse models. Thus, transgenic overexpression of CK2α in mammary glands causes mammary gland tumors with upregulated β-catenin and c-myc protein levels and NF-κB reporter activity [158]. In addition, CK2α activity and protein are elevated in carcinogen-induced mammary tumors in rats and mice [158,162].

CK2 inhibition alters cellular processes and blocks important signaling cascades. Thus, CK2 inhibition and knockdown of *CK2* lead to decreased cell numbers due to G2/M or G0/G1 arrest, apoptosis or senescence [44,153,154,163,164,165]. CK2 inhibition and knockdown of *CK2* also lead to changes in cell morphology, migration and invasion [153,154]. In addition, unbalanced expression of *CK2* genes promotes epithelial to mesenchymal transition in cell lines [13]. Importantly, *CK2α/α’* downregulation in triple negative (SUM-149) and *HER2* negative (MCF-7L) breast cancer cell lines resulted in increased apoptosis [155]. In addition, *CK2α/α’*downregulation lead to decreased clonal survival in triple negative SUM-149 and MDA-MB-231 [155]. These suggest that CK2 could be a therapeutic target even in triple negative breast tumors.

CK2 could act through several signaling pathways and mechanisms in breast cancer such as NF-κB, JAK/STAT, MAPK, Akt/MTOR, SIRT6, and miRNA expression [5,44,153,154,166,167]. Intriguingly, *CK2α* is among the targets of miR-125b, a miRNA that is decreased in breast tumor tissue. miR-125b inhibition promotes proliferation and reduced anchorage-independent proliferation [159]. 

CK2 also has links to breast cancer relevant proteins. For example, levels of CK2α and ERα proteins show a positive correlation in human breast cancer samples, human breast cancer cell lines and tumors from DMBA-treated rats [161]. Intriguingly, estrogen increases CK2α transcript and protein levels in an ERα-dependent manner through ERE sites in the *CK2α* promoter [161]. In turn, CK2α upregulation leads to increase proliferation, migration and anchorage-independent proliferation, and to PML degradation and AKT activation [161]. Additionally, CK2 phosphorylates PR, and this phosphorylation is necessary for anchorage-independent proliferation and expression of specific PR target genes [168].

Integration of CK2 inhibitors into treatment of breast cancer: The most likely clinical usage of CK2 inhibitors in the near future will be in the setting of hormone refractory or triple negative metastatic breast cancer. Thus far, there have not been dedicated breast cancer studies in humans but the pre-clinical data suggests strong potential for this approach. Thus, preclinical studies show that CK2 inhibitors reduce tumor burden. CX-4945 reduces tumor growth in orthotopic xenograft mouse models of breast cancer but did not affect mouse body weight or lead to overt toxicity [44]. Interestingly, CK2 activity decreased in tumors from a breast cancer xenograft model (MCF-7 cells) treated with dexamethasone, a chemosensitizer for breast cancer [169].

Importantly, CK2 inhibition has an effect in triple negative breast cancer xenograft models. Thus, nanocapsulated CK2α/α’ siRNA treatment reduces tumor growth in a mouse xenograft model correlating with reduced proliferation rates, but did not affect mouse body weight [155]. In addition, CX-4945 reduced IL-6 expression in a triple negative inflammatory breast cancer patient, in a xenograft model and triple negative breast cancer cell lines [170].

CK2 inhibition could help prevent resistance to anti-tumor drugs in breast cancer. Thus, overexpression of CK2α inhibits tamoxifen induced-senescence [165]. DMAT reduced cell numbers, increased apoptosis and changed morphology of tamoxifen-resistant breast cancer cells more efficiently than anti-estrogen sensitive MCF-7 parental line [171]. However, there were no differences in the protein levels of CK2α, CK2α and CK2β in tamoxifen-resistant versus nonresistant cells [171]. In contrast, breast cancer cell lines resistant to high levels of the antineoplastic agent VP-16 had CK2α transcript and protein elevated correlating with increase levels of phospho-topoisomerase IIα [172]. All together these data indicate that CK2 inhibitors could be used as potential therapeutics for triple negative and anti-estrogen resistant breast cancer.

##### Renal Cell Carcinoma

Renal cell carcinoma is a highly vascular cancer, which is responsible for approximately 14,000 deaths per year in the USA (out of approximately 61,000 cases). Clear cell carcinoma is the most common subtype of renal cell carcinoma and it is frequently accompanied by loss of expression of the Von Hippel-Lindau (VHL) gene which is a tumor suppressor gene that suppresses hypoxia-inducible factor (HIF) resulting in down-regulation of vascular growth factor (VEGF) production. When VHL expression is lost either through inherited or sporadic mutations, marked rise in VEGF and PDGF occurs which is major factor promoting growth and survival of renal cell carcinoma. Chemotherapy and radiation therapy have minimal benefit in advanced renal cell carcinoma; however, survival is extended by treatment with tyrosine kinase inhibitors targeting VEGF, MTOR inhibitors, and immunotherapy. There is unmet need for patients who progress on these therapies.

Rationale for CK2 inhibition in renal cell carcinoma: There is strong rationale for exploring CK2 inhibition in renal cell carcinoma. Renal cell carcinoma (RCC) samples show high expression of CK2α, *CK2α’* and *CK2β* transcripts [65,173,174]. Higher *CK2α* transcript expression correlated with higher grade and stage and with rate of metastasis [173,174]. Higher *CK2α’* transcript expression correlated with higher grade [173]. High *CK2α* transcript levels were a strong indicator of a poor overall survival, disease specific survival and progression free survival (*n* = 96) [173].

RCC samples show high CK2α, *CK2α’* and *CK2β* protein levels [173,175] and high levels of CK2 activity [173,176]. Importantly, in half of the samples there is unbalanced CK2α-α’/CK2β ratio that is due to increased CK2α-α’ expression and/or decreased CK2β expression. High nuclear CK2α staining was a strong indicator of a poor overall survival, disease specific survival and progression free survival (*n* = 40) [173]. In one study, the increase in CK2 proteins in RCC samples did not correlate with increased transcript levels; indeed, transcript levels decreased 1.5–16 times [175]. This is in contrast with the microarray data presented above.

CK2 can regulate VHL directly. CK2 inhibition stabilizes VHL, and therefore decreases HIF levels, although the mechanism is not yet defined [177,178]. CK2 phosphorylates VHL specifically at 3 N-terminal serine residues and stabilizes it [177,179]. Mutation of these 3 serine residues prevents the N-terminal protease cleavage that seems required for further proteasomal degradation of VHL [178]. However, mutation of the 3 serine residues only increased VHL half-life by 30% [177,178]. This suggests that additional mechanisms could be contributing to the increased VHL stability by CK2 inhibition. Importantly, expression of this mutation of these 3 serine residues delayed tumor onset by 6 weeks in a xenograft model [179].

Importantly, in VHL-deficient cells, CK2 inhibition results in decreased cell numbers and cell survival, correlating with decreased phosho-Akt and phosho-p21 and with increase phosho-p38 MAPK [173,175]. Together, these demonstrate a potential role for CK2 as a therapeutic target in renal cell carcinoma.

Integration in clinical care of renal cell carcinoma: The most active areas of clinical investigation in renal cell carcinoma of late include targeted drugs and immunotherapy. However, there remains an unmet need for patients who progress after use of these therapies. CK2 inhibition would be worth exploring in these patients and perhaps as well as a combination therapy with currently approved agents.

### 2.2. Hematological Malignancies

There has been a dramatic increase in new therapies for lymphomas and myeloma, and it may be harder for CK2 inhibitors to find niche in this setting. However there is rationale for use in these cancers (see below). In particular, acute leukemia has a greater need for new options since progress has been slower in this area.

#### 2.2.1. Leukemia

There is good evidence for a role for CK2 in acute myeloid leukemia (AML), acute lymphoid leukemia (ALL), and in chronic lymphocytic leukemia (CLL).

Rationale for CK2 inhibition: CK2 protein levels and activity are also deregulated in leukemias. B-ALL cell lines and primary B-ALL cells show upregulation of CK2 activity and CK2α and CK2α’ protein levels [180,181]. Primary T-ALL cells, show increased CK2 activity and CK2α and CK2β protein levels [182]. Primary AML cells and leukemia cell lines show increased levels of CK2 activity and CK2α protein [183,184]. Importantly, in patients with AML, high CK2α protein levels were a predictor of decreased overall and disease-free survival [183]. Primary CLL cells show increased CK2 activity and CK2α and CK2β proteins [185]. In summary, oncomine transcript expression and CK2 protein expression do not always correlate.

CK2 inhibition with CX-4945 resulted in increased apoptosis in B-ALL cell lines and primary B-ALL cells but, importantly, not primary normal bone marrow cells [180]. CK2 inhibition with CX-4945 resulted in decreased proliferation [186]. Two potential mechanisms have been proposed for the effect of CK2 inhibition in B-ALL: decreased PTEN and phospho-PTEN levels [180] and decreased expression of target genes of the tumor suppressor gene ikaros [186]. Importantly, xenograft models of primary B-ALL cells and cell lines, show that CX-4945 inhibits leukemia cell growth and increased mouse survival [186].

CK2 inhibitors TBB and DRB decreased cell viability in primary T-ALL cells while normal T-cells were unaffected [182]; CX-4945 also decreased cell viability [187]. CK2 overexpressed in T ALL cell lines and this correlates with increased Notch1 and myc activity in these cells. CX-4945 inhibits CK2 activity and has pro-apoptotic effect on these cells. CX-4549 promotes proteosomal degradation of Notch1 and decreases myc transcripts in the cells. Myc is downstream of Notch, hence CK2 inhibition could be an mechanism to inhibit myc. The target of CK2 action may be PTEN as CK2 can phosphorylate PTEN [32], CK2α overexpression correlates with PTEN phosphorylation in T-ALL primary cells, inhibition of CK2 led to increased PTEN activity, and subsequently decreased Akt phosphorylation [182,187]. CX-4945 decreased tumor growth in a xenograft model [187].

In AML cell lines, overexpression of CK2α leads to decreased proportion of cells in G0/G1 while inhibition of CK2 with apigenin, K27 and CX-4945 or with CK2α siRNA resulted in increased apoptosis [183,184]. Importantly, normal bone marrow cells were almost unaffected by apigenin [183]. In addition, CK2 inhibitors or CK2α/β siRNA sensitized AML cells to daunorubicin, an AML chemoterapeutic agent [184].

In CLL, CK2 inhibition with TBB and DRB led to decreased cell viability while leaving normal T and B cells unaffected [185]; CX-4945 also decreased cell viability [188]. Similar to ALL, primary CLL cells show phospho-PTEN upregulation, and *CK2* knockdown or inhibition decreased phospho-PTEN and PTEN expression [185].

Integration of CK2 inhibitors in treatment of leukemias: CIGB-300 promotes activation of the tumor suppressor PTEN and abrogates PI3K-mediated downstream signaling in CLL cells. In accordance, CIGB-300 decreases the viability and proliferation of CLL cell lines, promotes apoptosis of primary leukemia cells and displays antitumor efficacy in a xenograft mouse model of human CLL [187].

These experiments indicate a potential role for CK2 as a target for therapy in leukemia. Researchers have determined that two Phase 1 drugs (CX-4945 and JQ1) can work together to efficiently kill T-cell acute lymphoblastic leukemia cells while having minimal impact on normal blood cells. Despite treatment improvement, T-cell leukemia remains fatal in 20 percent of pediatric and 50 percent of adult patients. Both CX-4945 and JQ1 are currently in clinical trials as single agents to treat solid and hematological cancers. Based on a recent in vitro study it has been suggested that the combination treatment of CX-4945 and JQ1 could be an effective strategy to refractory/relapsed T-cell leukemia.

#### 2.2.2. Non-Hodgkin Lymphoma (NHL)

Many new agents are emerging for treatment of NHL, but there remains a strong need for new therapies. There are many variants of NHL so our discussion can only highlight specific findings relevant to CK2. T cell lymphomas are particularly difficult to treat.

Rationale of CK2 inhibition: Interestingly, overexpression of CK2α in lymphocytes of transgenic mice leads to T cell lymphoma [33,189,190]. A recently published study demonstrated increased CK2α and CK2β protein levels by immunoblot in follicular lymphoma, Burkitt’s lymphoma, and DLBCL, and in lymphoma cell lines [191].

Integration of CK2 inhibitors in treatment of lymphomas: Pharmacological inhibition of CK2 activity with CX-4945 led to dose-dependent increase in apoptosis in both Burkitt’s lymphoma and DLBCL cell lines [191]. In contrast, normal peripheral blood mononuclear cells were not affected by CX-4945. These data indicate a role of CK2 in NHL, and suggest that CK2 inhibitors could be used to diminish the survival of NHL tumor cells.

#### 2.2.3. Myeloma

The therapeutic options for multiple myeloma have expanded dramatically in recent years. However, this remains a very important malignancy, which is ultimately fatal in most cases. Hence, new options for therapy are still needed.

Rationale for CK2 inhibition: CK2α protein levels and CK2 kinase activity are increased in plasma cells from patients with multiple myeloma and in cell lines [192,193], and higher CK2α and CK2β staining in multiple myeloma tissues [192]. Furthermore, CK2 inhibition with TBB, IQA, a TBB-derivative K27 (2-amino-4,5,6,7-tetrabromo–1H-benzimidazole) and apigenin [194] led to decreased viability and increased apoptosis of myeloma cells. This indicates a role for CK2α in cell survival in myeloma [193,194,195]. CK2 inhibitors could be acting through decreasing NF-κB activation and transactivation activity [193]. CK2 inhibitors also decrease the endoplasmic reticulum (ER)-stress response leading to increased apoptosis [196]. The ER-stress/unfolded protein response is need for myeloma cells survival, due to the fact that myeloma cells produce abnormally large amounts of protein antibodies.

Integration of CK2 inhibitors into treatment of myeloma: CK2 inhibitors synergize with melphalan, the conventional chemotherapeutic agent used in myeloma treatment, to increase cytotoxicity [193]. CK2 inhibitors have an additive effect to geldanamycin, an antitumoral drug, to increase apoptosis [196]. Therefore, CK2 inhibitors may increase sensitivity (i.e., decrease dosage) to chemotherapy for myeloma [193].

## 3. Conclusions

CK2 is overexpressed in many cancers and often overexpression is associated with worse prognosis, although the opposite may be true in some cancer types as reviewed above [19]. CK2 can be used as a diagnostic and prognostic marker in certain malignancies, such as prostate cancer [19,137]. However, we the potential for CK2 expression as a prognostic and diagnostic marker could be greater as only a few studies analyze all three CK2 proteins, CK2 activity and localization. In addition, only a few studies analyze levels of CK2αP transcript that, as it was reviewed above, in some cancers are proposed as a prognostic marker. Analysis of all these parameters in future studies in cancer will realize the potential for CK2 as a diagnostic and prognostic marker. The mechanisms underlying the increases in CK2 transcript and protein levels are still unknown in many cancer types, although gene dosage alterations, epigenetic mechanisms and post-translational regulation have been proposed. In addition, in some cancers there are differences in CK2 activity without changes in levels of CK2α expression [93,144], suggesting additional post-translational mechanisms. Importantly, CK2 protein localization in the nucleus is found in a number of tumors, in some cases correlating with clinical parameters. This suggests that phosphorylation of nuclear target proteins is important for CK2’s role in cancer. However, we know less about the nuclear targets of CK2 during tumorigenesis compared to the cytoplasmic targets.

Individual knockdown of the *CK2* genes result effects on cellular processes, signaling pathway activation and gene expression that are qualitatively different in diverse tumors [37,38]. Therefore it will be important to develop CK2 inhibitors that could target specifically the monomeric or tetrameric complexes [197]. The development of specific inhibitors of the different CK2 forms for clinical use is being paralleled by targeted drug delivery methods, such as nanocapsules that are targeted to cancer cells [79].

CK2 has emerged as a potential anticancer target. As discussed above, an ample variety of cell-permeable CK2 inhibitors have been developed, and two of these CX-4945 and CIGB-300 have made into preclinical and clinical trials. This review shows how these inhibitors are already being employed in phase I/II trials in certain malignancies like lung, head and neck cancer, cholangiocarcinoma, cervical cancer and multiple myeloma with promising results for the future. With respect to the toxicity observed so far in clinical trials, CIGB-300 was fairly well tolerated in clinical trial of cervical cancer. The most frequent local events were pain, bleeding, hematoma and erythema at the injection site. The systemic adverse events were rash, facial edema, itching, hot flashes and localized cramps. CX-4945 was also fairly well tolerated in a phase 1 trial at MD Anderson cancer center involving patients with various advanced solid tumors and multiple myeloma. Diarrhea and hypokalemia were the dose limiting toxicities, and these toxicities were reversible with drug discontinuation, antidiarrheal use, and potassium supplementation.

These data also gives future perspective on how these two inhibitors can potentially be deployed for further clinical studies. They can be used as a single agent approach, like other signal transduction protein inhibitors in some cancers. They can also be combined with other signal transduction inhibitors, as CK2 regulates several signaling pathways that are key for tumorigenesis. They can be combined with chemotherapy or radiation therapy to prevent repair of DNA damage and increase cancer cell death. They can potentially be used either independently or in combination with other treatment modalities like immunotherapy and even with other Phase 1 drugs like JQ1. These two inhibitors have shown to enhance anti-proliferative effects as well as overcome resistance to the established chemotherapeutic agents, requiring much lower dosage, thus also possibly decreasing the toxic side effects, though not yet studied. CK2 inhibitors are also highly promising for specific use in HPV-related cancers and perhaps incorporation with other therapies for hematological malignancies.
